# Multisite Validation of Cryptococcal Antigen Lateral Flow Assay and Quantification by Laser Thermal Contrast

**DOI:** 10.3201/eid2001.130906

**Published:** 2014-01

**Authors:** David R. Boulware, Melissa A. Rolfes, Radha Rajasingham, Maximilian von Hohenberg, Zhenpeng Qin, Kabanda Taseera, Charlotte Schutz, Richard Kwizera, Elissa K. Butler, Graeme Meintjes, Conrad Muzoora, John C. Bischof, David B. Meya

**Affiliations:** University of Minnesota, Minneapolis, Minnesota, USA (D.R. Boulware, M.A. Rolfes, R. Rajasingham, M. von Hohenberg, Z. Qin, E.K. Butler, J.C. Bischof, D.B. Meya);; Makerere University, Kampala, Uganda (R. Rajasingham, R.K. Kwizera, D.B. Meya);; Mbarara University of Science and Technology, Mbarara, Uganda (K. Taseera, C. Muzoora);; G.F. Jooste Hospital, Cape Town, South Africa (C. Schutz, G. Meintjes);; University of Cape Town, Cape Town (C. Schutz, G. Meintjes)

**Keywords:** cryptococcal meningitis, Cryptococcus spp., fungi, cerebrospinal fluid, cryptococcal antigen, lateral flow immunochromatographic assay, cryptococcal antigen latex agglutination, culture, India ink microscopy, sensitivity, specificity, diagnostic techniques, validation studies, point-of-care systems, HIV, laser thermal contrast measurement, Uganda, South Africa

## Abstract

Cryptococcal meningitis is common in sub-Saharan Africa. Given the need for data for a rapid, point-of-care cryptococcal antigen (CRAG) lateral flow immunochromatographic assay (LFA), we assessed diagnostic performance of cerebrospinal fluid (CSF) culture, CRAG latex agglutination, India ink microscopy, and CRAG LFA for 832 HIV-infected persons with suspected meningitis during 2006–2009 (n = 299) in Uganda and during 2010–2012 (n = 533) in Uganda and South Africa. CRAG LFA had the best performance (sensitivity 99.3%, specificity 99.1%). Culture sensitivity was dependent on CSF volume (82.4% for 10 μL, 94.2% for 100 μL). CRAG latex agglutination test sensitivity (97.0%–97.8%) and specificity (85.9%–100%) varied between manufacturers. India ink microscopy was 86% sensitive. Laser thermal contrast had 92% accuracy (R = 0.91, p<0.001) in quantifying CRAG titers from 1 LFA strip to within <1.5 dilutions of actual CRAG titers. CRAG LFA is a major advance for meningitis diagnostics in resource-limited settings.

## Abstract 2

This assay is a major advance in the diagnosis of cryptococcal meningitis.

Cryptococcal meningitis is the most frequent form of meningitis among adults in sub-Saharan Africa (*1**–**4*) and accounts for 20%–25% of AIDS-related deaths in Africa (*5**–**7*). Although culture is the standard method for definitive diagnosis, detection of cryptococcal antigen (CRAG) in serum or cerebrospinal fluid (CSF) is used for presumptive diagnosis. CRAG screening in peripheral blood is also recommended for HIV-infected persons with CD4 cell counts <100/μL to reduce early deaths while receiving antiretroviral therapy (ART) (*8**–**10*). CRAG is usually detected by latex agglutination (CRAG latex), which has a sensitivity and specificity >99% (*11**,**12*). However, CRAG latex testing requires laboratory infrastructure and expertise, electricity, heat inactivation, cold-chain shipping, and refrigeration of reagents. Unfortunately, required infrastructure is usually unavailable in resource-constrained settings in which cryptococcal incidence is greatest (*5*). Thus, India ink microscopy is the primary diagnostic modality, despite having lower sensitivity (*12**–**14*).

In July 2011, a lateral flow immunochromatographic assay (LFA) (Immy, Inc., Norman, OK, USA) was approved by the US Food and Drug Administration for detection of CRAG in CSF and serum. This assay is a rapid diagnostic test that provides a definitive result in ≤10 min. With its low cost (currently $2/test for resource-limited settings and $5/test for high-income settings), shelf stability at room temperature, and ease of use, this rapid, point-of-care test might expedite diagnosis. Field testing data are needed for this assay.

Quantification of CRAG incidence can be determined by determining CRAG titer. Quantification is clinically useful because higher CSF CRAG titers are predictive of risk for death (*15**–**17*), and higher serum CRAG titers predict risk for immune reconstitution inflammatory syndrome (IRIS) (*18*). Titers can be determined by using CRAG latex or LFA, but these procedures are rarely used in resource-limited settings because of additional incremental cost per titer dilution. Thus, novel methods of quantification are needed.

In a multisite cohort of HIV-infected persons with suspected meningitis in sub-Saharan Africa, we compared CRAG LFA performance with that of traditional diagnostic tests. We demonstrated excellent diagnostic performance by the CRAG LFA in the laboratory and as a point-of-care bedside test. In addition, we demonstrated within this clinical cohort a novel method of CRAG titer quantification for an LFA by using laser thermal contrast measurement (*19*), enabling CRAG titer quantification without traditional serial dilutions.

## Materials and Methods

CRAG LFA was performed for 2 cohorts of prospectively enrolled HIV-infected persons with suspected meningitis: a 2006–2009 cohort of 299 hospitalized patients in Kampala, Uganda (*18**,**20*); and a 2010–2012 cohort of 533 hospitalized patients from G.F. Jooste Hospital, Cape Town, South Africa; Mbarara Hospital, Mbarara, Uganda (*16*); and Mulago Hospital, Kampala, Uganda (NCT01075152, www.clinicaltrials.gov). LFA was performed retrospectively with cryopreserved (−80°C) specimens collected before April 2011 and prospectively thereafter with point-of-care specimens. Persons with suspected meningitis provided written informed consent. Ethical approval was provided by each institutional review board and the Uganda National Council of Science and Technology.

### Diagnostic Testing

#### Quantitative CSF Fungal Culture

The fungal culture procedure in 2006–2009 used 10 µL of CSF cultured on Sabouraud dextrose agar (*12*). This method was insensitive for lower incidence infections. Thus, in 2010, the quantitative culture method was changed to a protocol using a 100-µL input volume of undiluted CSF culture and five 1:10 serial dilutions (*21**,**22*). Agar plates inoculated with CSF were incubated at 30°C for up to 14 days. The number of discrete colonies found at the highest dilution was multiplied by the dilution to give CFU/mL of CSF. Culture isolates were independently confirmed as *Cryptococcus neoformans* var. *grubii* by multilocus sequence typing (*23*).

#### CRAG Latex Agglutination

In 2006–2009, a qualitative CRAG latex assay (Meridian, Cincinnati, OH, USA) was performed in Kampala. Reactivity at a ≥1:2 dilution of CRAG was considered a positive result. Semiquantitative CRAG latex titers (*Cryptococcus* Antigen Latex Agglutination Test System; Immy, Inc.) were measured by 2-fold serial dilution on cryopreserved (−80°C) samples in Minnesota. Qualitative concordance between the 2 CRAG assays was imperfect (92%). Thus, results are presented separately.

In 2010–2013, the CRAG latex test kit (Immy, Inc.) was used in Uganda. Reactivity at a 1:2 dilution of CRAG was considered a positive result. CRAG titers were obtained for real-time specimens in Cape Town but for cryopreserved (−80°C) specimens in Mbarara and Kampala. Testing was performed without knowledge of results of alternative assays, except for culture. All laboratories participated quarterly in National Institutes of Health (Bethesda, MD, USA)–sponsored external quality assurance testing for fungal culture, India ink microscopy, and CRAG testing.

The 4 cryptococcal tests were not performed for all CSF samples because of insufficient sample volumes (most common), laboratory operating hours, or reagent supply chain difficulties. However, 794 (95%) of 833 samples were tested by ≥3 cryptococcal CSF tests performed prospectively, and 667 CSF samples were tested by LFA.

#### CRAG LFA

**Figure 1:**
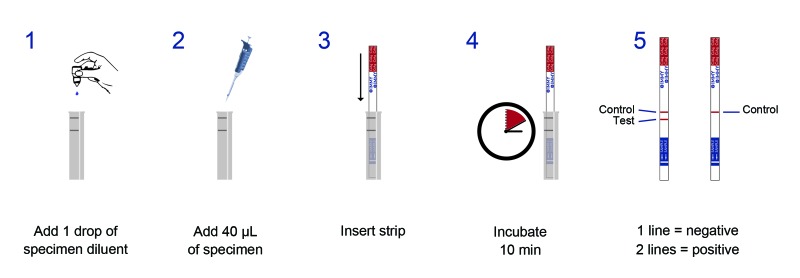
Five steps of the cryptococcal antigen lateral flow assay.

The LFA is a point-of-care dipstick test that uses gold-conjugated, monoclonal antibodies impregnated onto an immunochromatographic test strip to detect cryptococcal capsular polysaccharide glucuronoxylomannan antigen (CRAG) for all 4 *C. neoformans* serotypes (A–D) (*24**,**25*). If cryptococcal antigen is present in a specimen, suspended, gold-conjugated antibodies bind to the antigen. The gold–antibody–CRAG complex migrates by capillary action up the test strip, interacts with immobilized monoclonal antibodies against CRAG, and forms a red line. The LFA kit contains immunochromatographic test strips, positive controls, and assay diluent that can be stored at room temperature for ≤2 years. To perform the LFA, 1 drop of diluent (≈40 μL) is added to a container of 40 μL of patient specimen. The dipstick is inserted into the container and incubated at room temperature for 10 min (Figure 1).

All LFAs were performed according to manufacturer’s instructions by trained operators who had no knowledge of results of all other tests. For 2006–2010, cryopreserved CSF and plasma samples (−80°C) were retrospectively tested by LFA. Beginning in April 2011, the LFA was prospectively performed with CSF as a point-of-care test in the hospital ward and with serum (n = 346) and urine (n = 236) samples in Uganda collected on the day of lumbar puncture. Semiquantitative CRAG LFA titers were determined on cryopreserved samples by using a 2-fold serial dilution starting at 1:25. When samples qualitatively positive but negative at a 1:25 dilution, 2-fold dilutions starting at 1:2 were tested. CSF samples collected during 2010–2012 (n = 414) were qualitatively retested in Minnesota after storage at −80°C to determine inter-reader variability. Readers did not know prior LFA results.

#### Additional Diagnostic Testing

India ink microscopy was performed on sediment obtained after centrifugation of 1 mL of CSF. Gram staining, bacterial culture, and staining for acid-fast bacilli were prospectively performed for all specimens.

### Thermal Contrast Measurement of CRAG Titer

We used a novel method that provided quantification of LFAs in comparison with semiquantitative CRAG LFA titers by using the heat signature of laser-irradiated gold used in the LFA. To detect gold nanoparticles conjugated to monoclonal antibodies on the LFA line, the line was irradiated with a 0.01 W laser (532 nm, diode pumped; Millenia, Santa Clara, CA, USA) for 30 s, and temperature change (thermal contrast) was recorded with an infrared camera (A20; FLIR ThermoVision, Portland, OR, USA), as described (*19*). Three spots on each horizontal LFA line were irradiated and the average maximum temperature change was calculated. An antigen titer was calculated from the thermal contrast by using a calibration curve established by 2-fold serial dilutions of 3 specimens in triplicate with known CRAG LFA titers (R^2^ = 0.97). Thermal contrast quantification was performed for 115 positive and 58 visually negative CSF LFA dipsticks sequentially collected in Kampala during 2010–2011. Negative specimens were measured undiluted. Positive specimens were measured by thermal contrast at a 1:250 dilution to demonstrate a wide dynamic range, including specimens visually negative after dilution.

### Statistical Analysis

We compared diagnostic performance (sensitivity, specificity, and positive and negative predictive values) for each test versus a composite reference standard. The composite reference standard was defined as a CSF culture-positive (n = 459) or a culture-negative sample with ≥2 positive test results (e.g., India ink microscopy, CRAG latex, or CRAG LFA) and without an alternative etiologic explanation (n = 60). As such, no single positive test result (other than culture) could define the composite reference standard, enabling comparison of performance of the diagnostic tests, yet minimizing bias of the reference standard being defined solely by 1 test (*26*). When only 1 CSF test result was positive, serum cryptococcal antigenemia was used as a second qualifying positive test result (n = 6) to confirm the reference standard (tested during 2010–2012) as discrepant (*26*) on the basis of cryptococcosis pathogenesis in which antigenemia precedes culture-positive meningitis (*7**,**27**,**28*).

For thermal contrast measurements, Pearson correlation was used to compare predicted and actual LFA titers. To determine relative benefit from additional resources, we determined the cost of changing from India ink microscopy to CRAG LFA on the basis of the number needed to test (NNT) to detect 1 additional person with infected with *Cryptococcus* spp. We further modeled costs with a probabilistic sensitivity analysis by using TreeAge 2013 (TreeAge Software Inc., Williamstown, MA, USA) on the 95% CI of diagnostic test performance and published cryptococcal prevalence rates (*4*).

## Results

Among 832 persons with suspected meningitis during 2006–2012, a total of 525 (63%) had cryptococcal meningitis as defined per the composite reference standard. The 2 cohorts were similar in age, sex, and CD4 cell count (Table 1).

**Table 1:** Demographic characteristics and diagnostic tests performed with specimens from meningitis cohorts, Uganda and South Africa*

Characteristic	2006–2009, Retrospective	2010–2012, Prospective*		
Demographic	Kampala, Uganda	Kampala, Uganda	Mbarara, Uganda	Cape Town, South Africa
Location				
No. persons	299	354	142	37
Mean ± SD age, y	36 ± 8	35 ± 9	35 ± 11	37 ± 10
Male sex, no. (%)	168 (56)	174 (49)	87 (61)	22 (59)
CD4 cell count/μL median (IQR)	19 (7–38)	16 (7–69)	36 (14–74)	65 (43–97)
Diagnostic tests performed, no.				
CSF				
Quantitative culture	282	345	142	37
Latex agglutination	279	345	142	37
India ink microscopy	276	350	142	37
LFA	197	291	142	36
Serum				
Latex agglutination	85	34	NA	NA
LFA	NA	274	49	23
Plasma				
LFA	60	NA	NA	NA
Urine				
LFA	NA	185	51	NA

*IQR, interquartile range; CSF, cerebrospinal fluid; LFA, lateral flow immunochromatographic assay; NA, not available. †Prospective testing with LFA because point-of-care began in April 2011. Eighty LFAs were conducted on cryopreserved samples.

### LFA Diagnostic Performance

**Figure 2:**
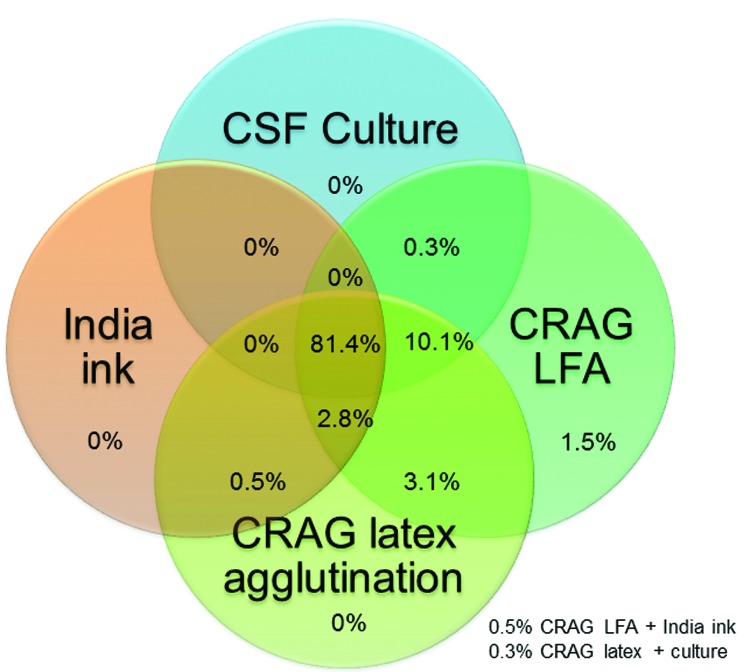
Venn diagram of distribution of 393 cryptococcal meningitis cases tested by 4 diagnostic assays, Uganda and South Africa. CSF, cerebrospinal fluid; CRAG LFA, cryptococcal antigen lateral immunochromatographic flow assay; India ink, India ink microscopy of 1 mL of concentrated CSF specimen. Numbers at the bottom right indicate 2 scenarios in which the Venn diagram does not overlap visually.

For 666 CSF samples available for testing, the LFA had a sensitivity of 99.3%, a specificity of 99.1%, a positive predictive value of 99.5%, and a negative predictive value of 98.7% (Table 2). Cryptococcal antigen testing by either latex or LFA was more sensitive than CSF culture. The 2006–2009 culture protocol used only 10 μL of CSF applied by a calibrated loop. This simple quantitative culture method was clinically useful but relatively insensitive (82.4%), and thus probably missed persons with low fungal incidence. The minimum growth on culture was 100 CFU/mL in 2006–2009. During 2010–2012, the culture protocol used 100 µL of CSF as the input volume and showed improved sensitivity (94.2%); the minimum growth detected was 10 CFU/mL. The median quantitative CSF culture grew 150,000 CFU/mL of CSF (interquartile range [IQR] 14,100–455,500 CFU/mL) in 2010–2012. The overlap of specimens with positive results for the 4 cryptococcal diagnostic assays is shown in Figure 2.

**Table 2:** Performance characteristics of cryptococcal diagnostic assays in persons with suspected meningitis, Uganda and South Africa*

Diagnostic test	No.	No. positive/no. tested (%)			
		Sensitivity	Specificity	PPV	NPV
CRAG LFA	666	435/438 (99.3)	226/228 (99.1)	435/437 (99.5)	226/229 (98.7)
CSF culture†	806	459/510 (90.0)	296/296 (100.0)	459/459 (100.0)	296/347 (85.3)
100-μL volume	524	309/328 (94.2)	196/196 (100.0)	309/309 (100.0)	196/215 (91.2)
10-μL volume	282	150/182 (82.4)	100/100 (100.0)	150/150 (100.0)	100/132 (75.8)
India ink microscopy	805	438/509 (86.1)	288/296 (97.3)	438/446 (98.2)	288/359 (80.2)
CRAG latex (Meridian)‡	279	176/180 (97.8)	85/99 (85.9)	176/190 (92.6)	85/89 (95.5)
CRAG latex (Immy)§	749	452/466 (97.0)	283/283 (100.0)	452/452 (100.0)	283/297 (95.3)

*PPV, positive predictive value; NPV, negative predictive value; CRAG, cryptococcal antigen: LFA, lateral flow immunochromatographic assay; CSF, cerebrospinal fluid. All samples tested by CSF CRAG LFA had false-positive results and did not have any other pathogen identified. However, serum or plasma samples were not available for testing for cryptococcal antigenemia to determine if the original result indicated enhanced detection. †Two quantitative CSF culture procedures were used in 2006–2009 (input volume 10 μL) and 2010–2012 (input volume 100 μL). ‡Meridian, Cincinnati, OH, USA. §Immy, Inc., Norman, OK, USA.

Three samples had false-negative results by LFA; all were retested. Of the 1 culture- positive sample with a negative result by LFA, it had a relatively low fungal incidence of 100 CFU/mL (Table 3). The 3 false-negative samples detected by LFA were previously cryopreserved CSF supernatant specimens, and a freeze/thaw artifact or error during storage cannot be excluded. During prospective performance, LFA detection of low fungal incidence in culture was not a systematic problem. Among 10 persons with culture incidence ≤100 CFU/mL in 2011–2012, LFA prospectively detected 10 of 10, CRAG latex detected 9 of 10, and India ink microscopy detected 3 of 10.

**Table 3:** Characteristics of CSF specimens with false-negative results by cryptococcal antigen lateral flow immunochromatographic assay, 2006–2009 cohort, Uganda and South Africa*

CSF culture, CFU/mL	India ink microscopy	CSF latex agglutination test dilution†	CSF CRAG LFA
100	–	1:2 Meridian; 1:1 Immy	–‡
0	+	1:2 Meridian; 1:1 Immy	–‡
0	+	1:2 Meridian; 1:1 Immy	–‡

*CSF, cerebrospinal fluid; CRAG, cryptococcal antigen; LFA, lateral flow immunochromatographic assay; CFU, colony-forming units of Cryptococcus neoformans; –, negative; +, positive. †Meridian, Cincinnati, OH, USA; Immy, Inc., Norman, OK, USA.  ‡Retrospectively performed with cryopreserved specimens.

Regarding LFA specificity, 8 CSF samples were positive only by CRAG LFA. We initially believed that these samples had LFA false-positive results (Table 4). Culture-positive cyyptococcal meningitis developed 6 weeks later in a person with a possible false-positive LFA result. A second person with a possible false-positive LFA result died of meningitis symptoms several weeks later, and a third person with a possible false-positive LFA result had cryptococcoma masses documented postmortem. Of 8 persons with CSF samples positive by LFA only (negative by culture, India ink microscopy, and CRAG latex), 6 had serum cryptococcal antigenemia. These 6 persons were classified as having true cryptococcal disease. Two persons in the 2006–2009 cohort did not have a peripheral blood specimen collected, and thus were conservatively classified as having false-positive results Their CSF specimens were tested for bacteria, herpes viruses, arboviruses, toxoplasmosis, syphilis, and tuberculosis but no alternative etiology was identified. If the 6 samples from persons without documented evidence of cryptococcal meningitis were considered as having false-positive results, the LFA specificity would decrease to 97% (226/234).

**Table 4:** Characteristics of CSF specimens positive only by cryptococcal antigen lateral flow immunochromatographic assay, Uganda and South Africa*

Cohort	CSF LFA titer	Serum CRAG LFA titer	Serum CRAG latex titer	Urine LFA result	Classification	Patient outcome
2006–2009	2	NA	NA	NA	LFA false positive	Unknown
	2	NA	NA	NA	LFA false positive	Unknown
2010–2012	2	512	1:8	–	Cryptococcal meningitis	Began ART; CSF culture-positive result; meningitis developed 6 weeks later
	±	2,048	2,048	–	Cryptococcal meningitis	Died after hospital discharge
	±	32	–	+	Cryptococcal meningitis	Died in hospital
	250	4	–	+	Cryptococcal meningitis	Began ART; minimum CD4 cell count 130 cells/μL
	8	8	128	–	Cryptococcal meningitis	Died after hospital discharge
	±/16†	4,096	2,048	+	Cryptococcal meningitis	Given fluconazole, 800 mg/d; seizure; died; cryptococcoma mass identified postmortem

*CSF, cerebrospinal fluid; CRAG, cryptococcal antigen; LFA, lateral flow immunochromatographic assay; –, negative’ NA, not available; ART, antiretroviral therapy; ±, discordant readings by 2 independent readers; +, positive. All test results for CSF culture, India ink microscopy, and CSF CRAG latex were negative. †Fresh specimen was CRAG latex and LFA negative. CRAG LFA titer of 16 on cryopreserved specimen. Repeat CSF testing results 2 weeks later for neurologic deterioration were positive and remained CRAG latex negative. Thermal contrast showed that all LFA strips had positive results with readings above background.

CSF CRAG LFA titers correlated with other quantitative measures, such as CSF culture (Pearson r = 0.58, p<0.001) and CRAG latex titers (r = 0.82, p<0.001). When comparing CRAG titers, LFA median titer was 2.5-fold higher (IQR 1.25–5-fold) than CRAG latex titer (e.g., CSF with a CRAG latex titer of 1,024 had an LFA titer of 2,500). This LFA enhancement of higher titers relative to CRAG latex was more prominent at lower titers. For CRAG latex titers ≤256, LFA titers were a median 5-fold (IQR 2.5–10-fold) higher than CRAG latex titers, whereas for CRAG latex titers ≥2,048, LFA titers were a median 2.5-fold (IQR 0–4.4-fold) higher.

LFA readings were concordant for 98.8% (409/414) of prospective samples retested after storage by an independent reader. All discordant results were negative initially on fresh samples read under indirect sunlight in Uganda, but 4 were weakly positive and 1 was strongly positive when retested after cryopreservation in the United States and read under bright fluorescent lighting.

### India Ink Microscopy

Sensitivity of India ink microscopy was the lowest (86%) of any test and highly dependent on fungal burden. Sensitivity decreased to 42% (19/45) among persons with CSF cultures having <1,000 CFU/mL. Overall, 1 of 7.2 cryptococcal diagnoses was missed by India ink microscopy (negative predictive value of 80%; (95% CI 76%–84%). If India ink microscopy had been the only diagnostic test used, 8.8% of meningitis cases in Uganda would have been misdiagnosed. Among persons in Uganda who had India ink microscopy–negative results, *Cryptococcus* spp. remained the most common pathogen (20%). The NNT by CRAG LFA was 5.1 (95% CI 4.1–6.5) per additional cryptococcal diagnosis.

By replacing India ink microscopy with CRAG LFA for meningitis diagnostics, we found that overall NNT was 11.4 (95% CI 9.1–15.1) among HIV-infected persons in Uganda with suspected meningitis, and LFA cost per additional diagnosis was US $22.83 (95% CI $18.22–$30.28) for LFA supplies, excluding savings for India ink microscopy costs and inappropriate medications prescribed (e.g., ceftriaxone US $2/day, international wholesale price) (*29*). When we extrapolated cryptococcal prevalence rates in eastern and southern Africa (37% of meningitis cases; 95% CI 35%–39%) (*1**–**4*), the NNT was 20.2 (95% CI 13.3–40.6) at a cost of $40 (95% CI $27–$80) for LFAs per additional diagnosis. In middle- and high-income countries in which laboratory labor is the primary cost component, LFAs would have lower costs than India ink microscopy or CRAG latex.

### Peripheral Blood and Urine Samples

Among serum samples tested, sensitivity was 98.3% (114/116) for serum CRAG latex and 99.6% (239/240) for serum LFA among persons with cryptococcal meningitis. Serum LFA specificity was 92% (98/106) for cryptococcal meningitis. The correlation between serum CRAG latex and serum LFA titers was r = 0.87 when tested independently (p<0.001). Of 60 plasma specimens retrospectively tested from the 2006–2009 cohort, LFA sensitivity was 100% (specificity unavailable). Plasma LFA titers and serum CRAG latex titers showed a correlation (r = 0.89, p<0.001), but median LFA titer was 3.3-fold (IQR 2.3–4.3-fold) higher than CRAG latex titer. Strength of the correlation was less between CSF LFA titer and either serum LFA titer (r = 0.54, p<0.001) or plasma LFA titer (r = 0.15, p = 0.30). LFA for urine had a sensitivity of 97% (151/156) and a specificity of 85% (68/80) for cryptococcal meningitis. Among urine specimens from persons with false-positive results, 5 of 7 had antigenemia detected by CRAG LFA of serum.

### Thermal Contrast

**Figure 3:**
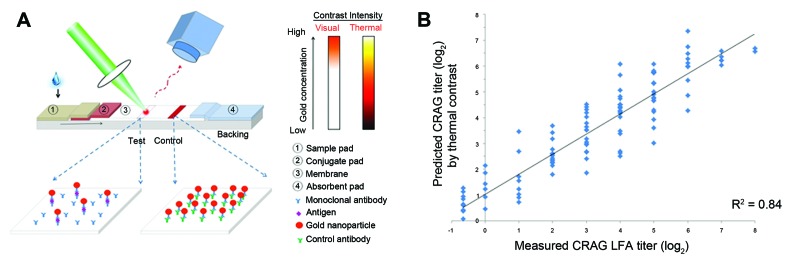
A) Prediction of cryptococcal antigen titer based on laser thermal contrast measurement and concept of lateral flow immunochromatographic assay (LFA) thermal contrast measurement in which a laser irradiates the test line in the LFA (*19*). The test line is formed by gold–monoclonal antibody–antigen sandwich complex with a monoclonal antibody affixed at the test line. When irradiated by a green laser (532 nm), any gold present absorbs light from the laser and generates heat in direct proportion to the amount of gold (and thereby antigen) present at the test line. This temperature change can be measured by using an infrared camera. B) Association of measured semiquantitative LFA cryptococcal antigen (CRAG) titer starting at a 1:250 dilution by the predicted CRAG titer based on thermal contrast measurement. Measurements on the negative portion of the x-axis are beyond the visual range when specimens were diluted 1:250, yet still detectable by thermal contrast. The Pearson correlation coefficient was r = 0.91 (p<0.001, R^2^ = 0.84) among 115 positive specimens quantified. A total of 58 LFA CRAG–negative specimens established background levels of heat radiation.

We developed a novel method to quantify CRAG titers from 1 LFA strip by using laser thermal contrast measurement to convert a qualitative LFA into a quantitative assay. The technique measures the change in temperature of gold nanoparticles when laser irradiated, and in comparison with a calibration curve, we predicted CRAG titer (*19*). Among 115 CSF LFA-positive samples and 58 negative samples, the predicted titer by thermal contrast correlated (r = 0.91, p<0.001) with actual semiquantitative titer by LFA, as performed by traditional 2-fold serial dilutions (Figure 3). Overall, 92% of predicted CRAG titers by thermal contrast were within 1.5 dilutions of the actual semiquantitative titer when measured by serial dilution.

We tested specimens at a 1:250 dilution and detected visually negative specimens (after dilution) by thermal contrast, which demonstrated enhanced sensitivity. All 58 undiluted negative specimens from persons without *Cryptococcus* spp. infection remained negative by thermal contrast, which demonstrated maintained specificity. Thermal contrast removed the subjectivity of borderline, equivocally positive LFA results. Furthermore, because gold is inert, LFA can be subjected to repeated thermal contrast measurements without major changes in measurement. Readings obtained in LFAs were virtually identical (R^2^ = 0.99) at 2 weeks, 2 months, and 6 months. Thus, archiving of LFA samples for delayed reading is possible (e.g., to centralize laboratory processing for quality control).

## Discussion

This multisite study prospectively validated a new point-of-care CRAG LFA in a clinical setting among persons with suspected meningitis in sub-Saharan Africa. These results confirm that the CRAG LFA has ≥99% sensitivity and ≥99% specificity for CSF in the field. We demonstrated that a novel technique, laser thermal contrast, had 92% accuracy in quantifying CRAG titers from 1 LFA strip to within <1.5 dilutions of the actual CRAG titer by serial dilutions (R = 0.91, p<0.001). LFA performance was more sensitive than that of any other diagnostic test. Conversely, the worst performing test was India ink microscopy, which is the most common cryptococcal diagnostic test in Africa, despite missing 1 in 7 cryptococcal diagnoses and having only an 80% negative predictive value in our cohorts. In facilities with India ink microscopy testing capability, switching to CRAG LFA would result in 5% more meningitis patients with a correct diagnosis in eastern and southern Africa (*4*). In addition, the CRAG LFA now enables diagnostic testing anywhere for the most common cause of meningitis in Africa.

The enhanced LFA analytic sensitivity is reflected by median 2.5-fold higher titers observed for CSF with LFA than with CRAG latex, indicating that LFA is more sensitive to lower antigen levels. Increased sensitivity of the LFA became clinically apparent in CSF specimens from persons positive only by LFA who showed CRAG positivity in serum. We observed 6 such case-patients in the 2010–2012 cohort. Initially, these persons were considered to have false-positive results. However, culture-positive cryptococcal meningitis developed in some persons or these persons died. These findings suggest that early-stage cryptococcal meningitis might be missed by traditional diagnostics. If these cases represent early cryptococcal meningitis, they would argue for expansion of targeted screening for cryptococcal antigenemia in peripheral blood in the setting of unexplained meningitis or repeat testing.

Our study builds upon 5 recent studies validating LFA versus ELISA and latex agglutination in laboratory settings (*24**,**30**–**33*). Jarvis et al. validated the LFA in 62 persons with a history of cryptococcal meningitis within the preceding 2 years in South Africa but without any concurrent controls (*24*). At 2 US and 1 Australian reference laboratories, the CRAG LFA had 100% sensitivity and excellent specificity when used to test 18 persons (*31*), 17 persons (*32*), and 25 persons (*33*) with cryptococcal infections, including detection of *C. gattii* (*31**–**33*). Our study demonstrates excellent performance of LFA as a point-of-care assay.

A point-of-care assay has multiple practical advantages. First, time to diagnosis can be reduced. During prospective implementation, median time between lumbar puncture and reporting CRAG latex results was 4 h and 50 min in Kampala and >24 h in Cape Town. When LFA was performed at bedside, the result was known in ≤10 min. Second, this point-of-care test can be used in rural settings without laboratory infrastructure. Third, cryptococcal guidelines recommend control of intracranial pressure (*8**,**34**–**36*). However, manometers are unavailable in Africa. Knowing cryptococcal status beforehand enables intervention during lumbar puncture to therapeutically control intracranial pressure. In contrast, when the cryptococcal diagnosis is not known until after lumbar puncture, the opportunity to intervene to control intracranial pressure has been lost. We suggest CRAG LFA immediately before the lumbar puncture in HIV-infected persons (e.g., with serum, plasma, or fingerstick blood). If results are positive, a manometer could be prioritized for use or, if unavailable, 20 mL CSF could be empirically drained for management of intracranial pressure.

One impracticality of determining CRAG titers in low- and middle-income countries is cost. CRAG titers are useful for predicting survival and risk for paradoxical IRIS, identifying *Cryptococcus* spp. in persons receiving ART who had pre-ART cryptococcal antigenemia, and differentiating cryptococcal-IRIS from cryptococcal relapse (*16**,**20**,**28**,**37**,**38*). However, each CRAG titer dilution requires the same amount of reagent as the initial test and is labor-intensive. Thermal contrast might be a useful, low-cost technology to increase sensitivity of LFA rapid diagnostic tests and provide quantification of results. Although this possibility is currently a prototype concept, it is probably applicable to any gold nanoparticle–based LFA (e.g., for malaria, *Mycobacterium tuberculosis*–associated lipoarabinomannan, pneumococcus, testing during pregnancy). Future work to construct a handheld, mobile-linked device can leverage this technology to improve sensitivity and quantification of antigen levels for any gold nanoparticle–based LFA.

In conclusion, the CRAG LFA is a sensitive and specific point-of-care assay for diagnosis of cryptococcal meningitis and has particular applicability in resource-limited settings. Our experience suggests the LFA is more sensitive than current diagnostics and will enable detection of early-stage cryptococcal infection. In addition, the CRAG LFA is practical and may dramatically change the face of meningitis diagnostics worldwide.
